# Supplemental S1 fixation for type C pelvic ring injuries: biomechanical study of a long iliosacral versus a transsacral screw

**DOI:** 10.1007/s10195-015-0357-8

**Published:** 2015-05-31

**Authors:** Pooria Salari, Berton R. Moed, J. Gary Bledsoe

**Affiliations:** Department of Orthopaedic Surgery, Saint Louis University School of Medicine, 3635 Vista Avenue, 7th Floor Desloge Towers, St. Louis, MO 63110 USA; Department of Biomedical Engineering, Parks College of Engineering, Aviation and Technology, Saint Louis University, 3450 Lindell Boulevard, St. Louis, MO 63103 USA

**Keywords:** Iliosacral screws, Transsacral screws, Type C pelvic ring injuries

## Abstract

**Background:**

A single iliosacral screw placed into the S1 vertebral body has been shown to be clinically unreliable for certain type C pelvic ring injuries. Insertion of a second supplemental iliosacral screw into the S1 or S2 vertebral body has been widely used. However, clinical fixation failures have been reported using this technique, and a supplemental long iliosacral or transsacral screw has been used. The purpose of this study was to compare the biomechanical effect of a supplemental S1 long iliosacral screw versus a transsacral screw in an unstable type C vertically oriented sacral fracture model.

**Materials and methods:**

A type C pelvic ring injury was created in ten osteopenic/osteoporotic cadaver pelves by performing vertical osteotomies through zone 2 of the sacrum and the ipsilateral pubic rami. The sacrum was reduced maintaining a 2-mm fracture gap to simulate a closed-reduction model. All specimens were fixed using one 7.0-mm iliosacral screw into the S1 body. A supplemental long iliosacral screw was placed into the S1 body in five specimens. A supplemental transsacral S1 screw was placed in the other five. Each pelvis underwent 100,000 cycles at 250 N, followed by loading to failure. Vertical displacements at 25,000, 50,000, 75,000, and 100,000 cycles and failure force were recorded.

**Results:**

Vertical displacement increased significantly (*p* < 0.05) within each group with each increase in the number of cycles. However, there was no statistically significant difference between groups in displacement or load to failure.

**Conclusions:**

Although intuitively a transsacral screw may seem to be better than a long iliosacral screw in conveying additional stability to an unstable sacral fracture fixation construct, we were not able to identify any biomechanical advantage of one method over the other.

**Level of evidence:**

Does not apply—biomechanical study.

## Introduction

Pelvic fractures account for 1–3 % of all skeletal fractures and comprise a broad spectrum of injuries: from low-energy fractures in osteoporotic patients to high-energy disruptions of the pelvic ring [1, 2]. Type C pelvic ring injuries are vertically unstable due to complete disruption of the posterior arch [3–6]. This posterior injury is by necessity accompanied by a second injury site in the ring, commonly in the anterior arch of the pelvic ring, and consisting of disruption of the pubic symphysis, and ipsilateral and/or contralateral fractures of the superior and inferior pubic rami [3, 7, 8]. Posterior ring disruption is associated with high morbidity and mortality rates [9, 10]. As shown in multiple studies following treatment of the pelvic injury, residual deformity or associated injuries can create significant problems in functional recovery [11, 12]. Numerous investigators have found that displacement through the weight-bearing arch of the pelvis can lead to long-term problems of pain and inability to regain function and resume previous lifestyle [13–17]. Regardless of the exact location of the posterior disruption, early restoration of pelvic ring integrity is vital, and surgical management is thought to reduce long-term complications, such as malunion, nonunion, neurologic dysfunction, low-back pain, and gait abnormalities [4, 15–22].

Many surgical techniques have been described for fixation of the posterior pelvic ring injury, with iliosacral screw fixation into the first sacral body being in common practice [4, 8, 19, 23–26]. Single iliosacral screw fixation into the S1 vertebral body has been shown to be clinically unreliable for unstable type C vertically oriented sacral fractures [8]. Insertion of a second, supplemental, iliosacral screw into the S1 or S2 vertebral body has been widely used [27]. In 2006, Moed and Geer published data on series of patients reporting safe use of S2 screws. However, they raised the concern about using this type of screw in osteopenic patients and recommended its use only with good bony purchase after instrumentation [28]. More recently advocated is the use of a long iliosacral screw (extending from the external surface of the ilium to just short of the contralateral sacroiliac joint) or a transsacral screw (extending from the external surface of the ilium across the contralateral sacroiliac joint and exiting the ilium) [8, 29, 30]. To our knowledge, no biomechanical study has been performed to differentiate the effect of these two screw lengths on fixation construct stability in type C, zone 2 sacral fracture with a residual gap at the fracture site to mimic the clinical situation of a closed reduction in which an anatomic reduction of the sacral fracture is not attained.

The purpose of this study was to biomechanically compare the effect of a supplemental S1 long iliosacral screw versus a transsacral screw in an unstable type C vertically oriented zone 2 sacral fracture model [3, 5].

## Materials and methods

Ten embalmed cadaver pelves with intact, attached 4th and 5th lumbar vertebrae were harvested with ligamentous structures (including sacroiliac, sacrospinous, sacrotuberous, and symphyseal ligaments) and sacroiliac joint capsules kept intact. Using a GE Lunar Scanner (GE Healthcare, UK), dual-emission X-ray absorptiometry was performed on each specimen. All specimens were osteopenic, with a *T* score of ≤−1 [31]. Subsequently, a completely unstable and displaced type C pelvic ring injury with a zone 2 sacral fracture was created using the following steps:

1. The right superior and inferior pubic rami were osteotomized in a vertical fashion using an oscillating surgical power saw with a thin blade.
2. An ipsilateral vertical zone 2 sacral fracture was created by making a unilateral cut through the sacral neuroforamina using an oscillating surgical power saw with a thin blade.
3. The ipsilateral sacrospinous and sacrotuberous ligaments were transected to ensure complete disruption of the sacroiliac complex.

**Fig. 1:**
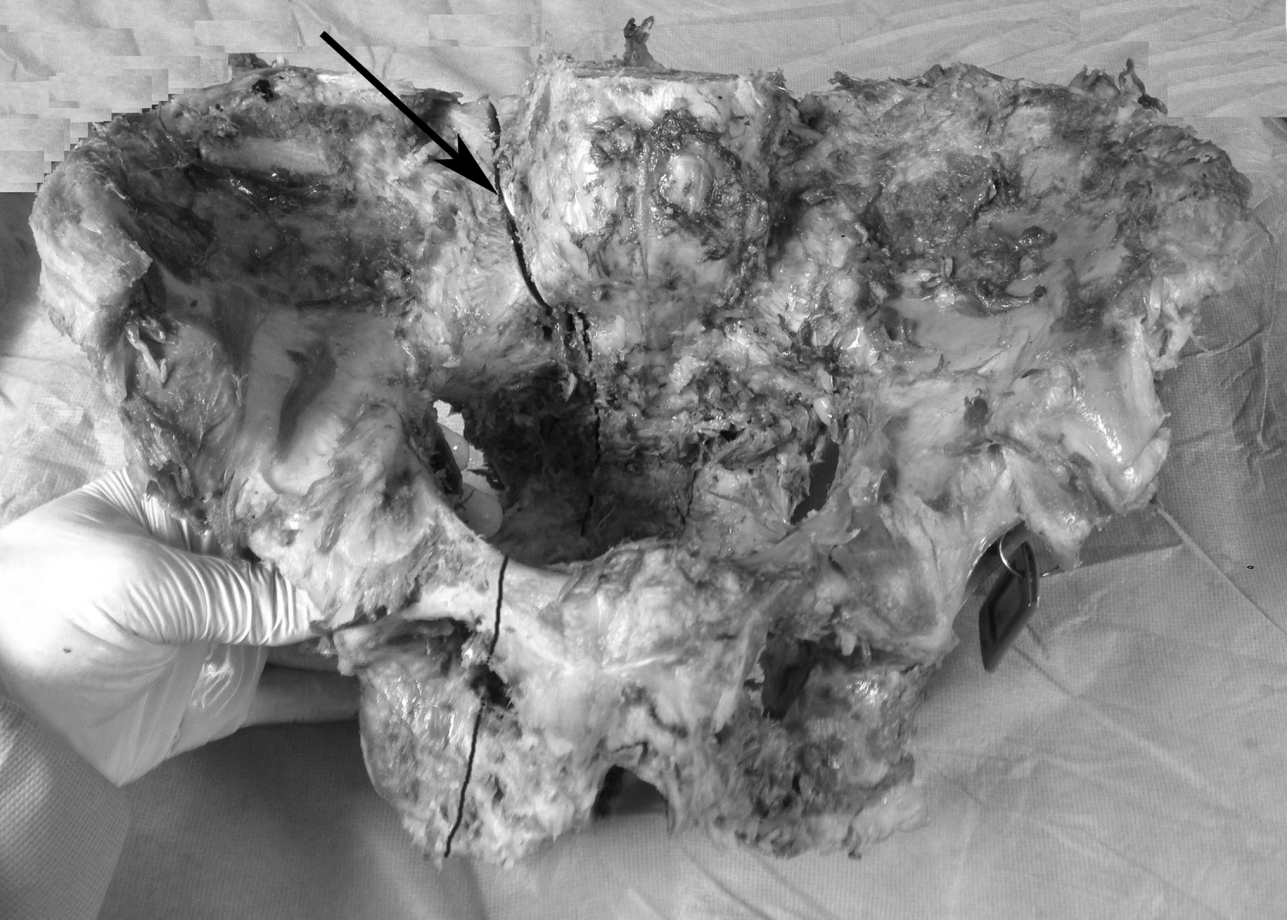
Pelvis showing the creation of an unstable type C, zone 2, vertically oriented injury. The *arrow* points to the 2-mm spacer used to create a fracture gap

**Fig. 2:**
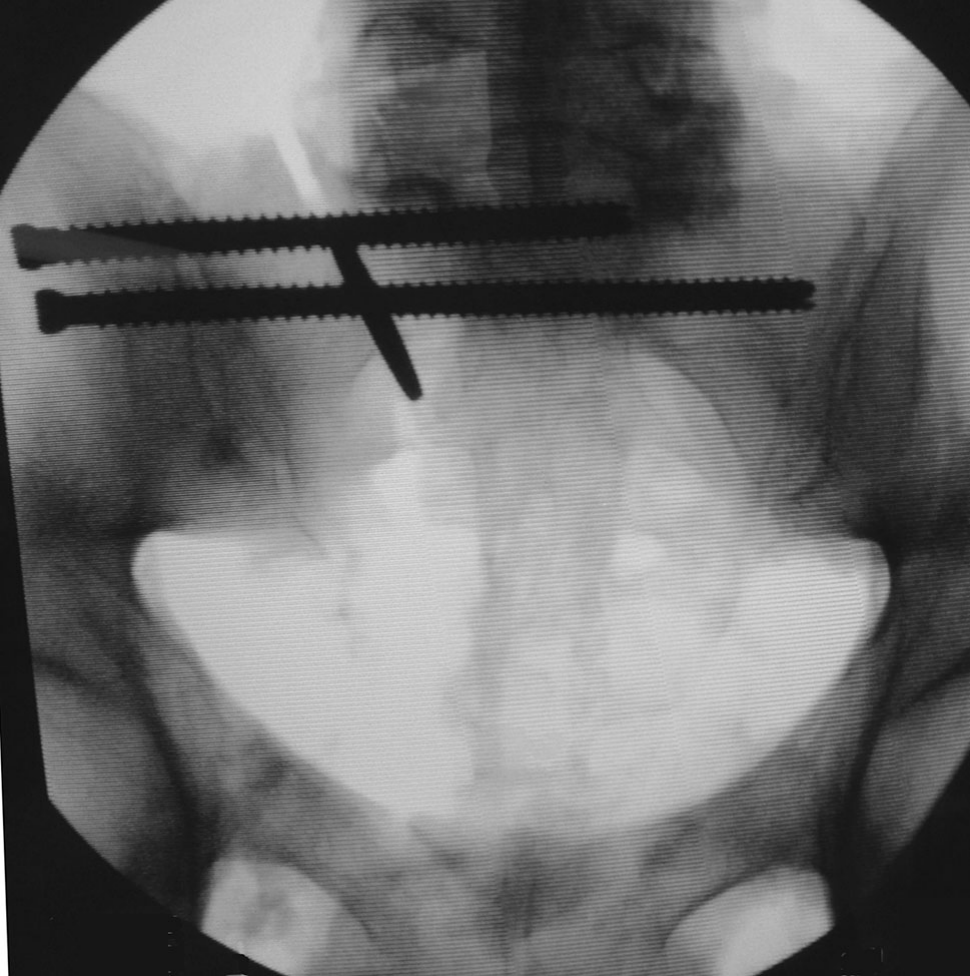
Fluoroscopic image of a pelvis instrumented in the iliosacral group. The spacer was removed after screw placement

**Fig. 3:**
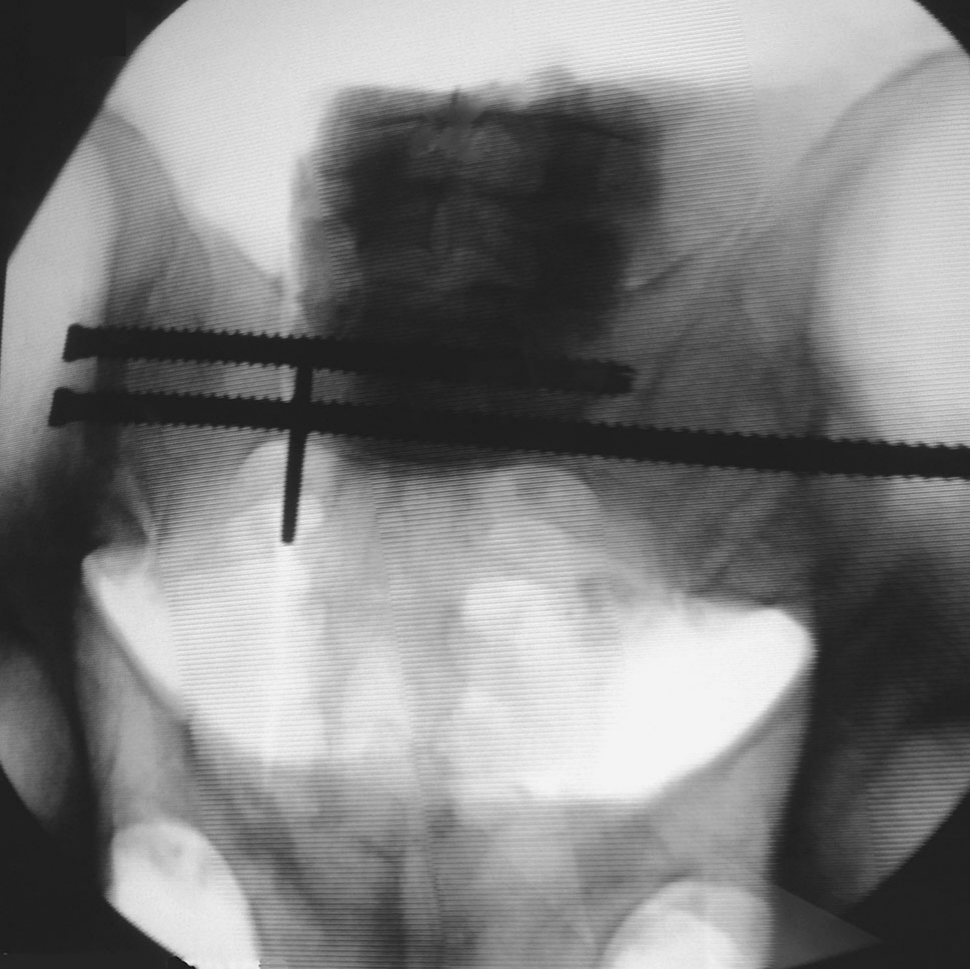
Fluoroscopic image of a pelvis instrumented in the transsacral group. The spacer was removed after screw placement

This simulated, completely displaced, vertical zone 2 sacral fracture was then reduced in distraction, maintaining a 2-mm fracture gap with a calibrated spacer (Fig. 1). Using fluoroscopic guidance, each specimen was then fixed using one standard-length 7.0-mm stainless steel cancellous fully threaded cannulated iliosacral screw (Zimmer, Inc., Warsaw, IN, USA) into the S1 vertebral body. Next, again using fluoroscopic guidance, a supplemental long iliosacral screw was then placed into the S1 body in five specimens (Fig. 2) and a supplemental transsacral S1 screw was placed in the other five specimens (Fig. 3). To ensure similarity of bone density between these two groups, specimens were matched based on *T*-score values. The pelves in the long iliosacral and transsacral groups had *T* scores that were not significantly different, with means of −2.28 (range −1.4 to −3.4) and −2.38 (range −1.1 to −4.1), respectively (*p* = 0.62; Mann–Whitney *U* test). The osteotomized ipsilateral superior and inferior pubic rami were not fixed in any of the pelvis specimens. After fluoroscopic imaging confirmed appropriate sacral fracture reduction and screw placement (Figs. 2 and 3), the spacer was removed to mimic the clinical situation of a zone 2 sacral fracture percutaneously fixed without compression.

**Fig. 4:**
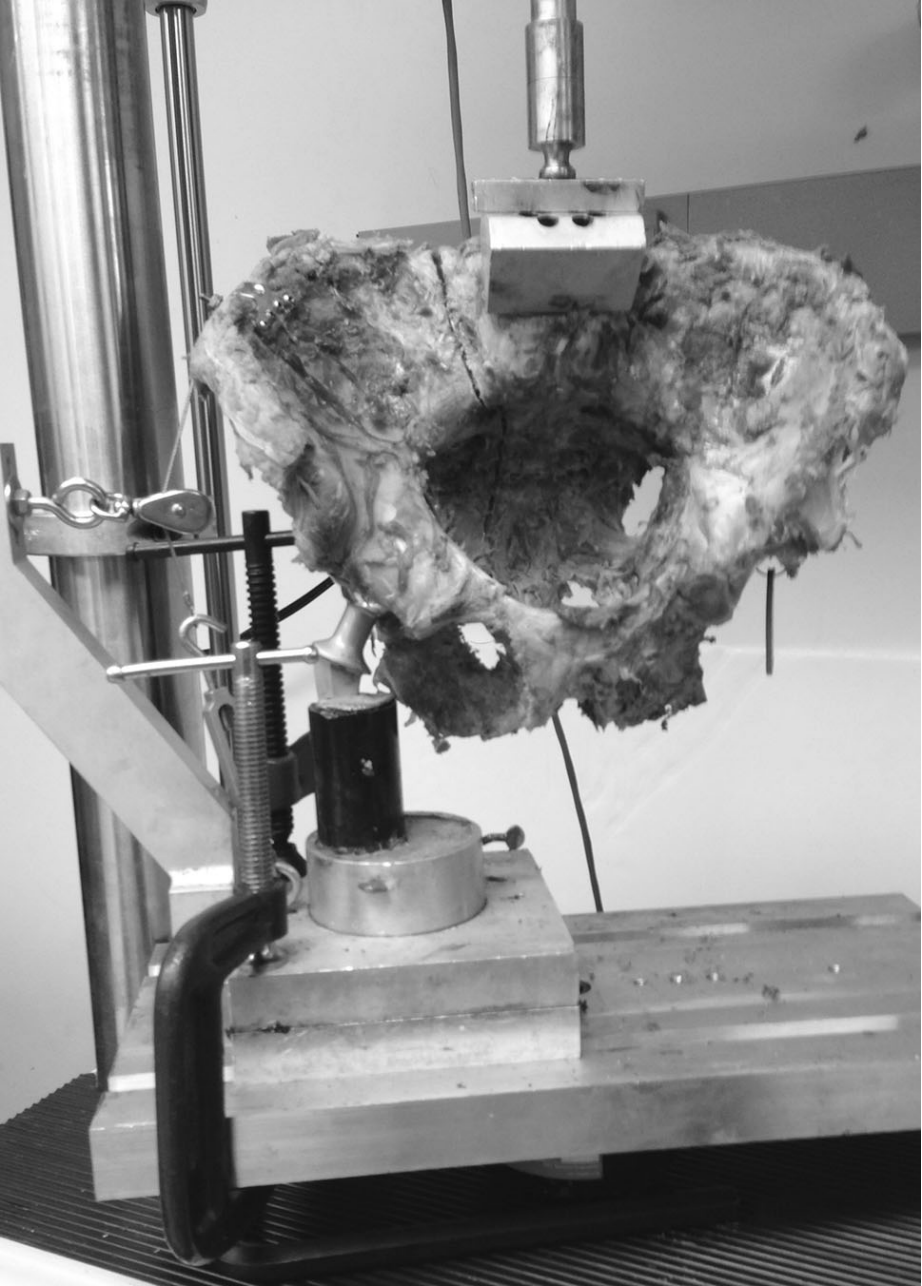
Pelvis loaded into the MTS machine using the single-limb-stance model

Using a previously described single-limb stance-testing model, each pelvis was mounted on a servohydraulic materials-testing system (MTS 858 Mini Bionix, MTS Systems, Inc., Eden Prarie, MN, USA) [32, 33]. The acetabulum on the ipsilateral side of the disrupted pelvis was fitted with a potted femoral arthroplasty component that was secured to the platform of the MTS machine with two large C clamps to prevent any side-to-side motion. A T plate was fixed to the ipsilateral iliac crest, and the pelvis was linked to a pulley system by a cable incorporated into the jig (Fig. 4). Then, the pelvis was loaded using the MTS hydraulic actuator, with the force being applied through a stainless steel ball-and-socket articulation attached to the superior endplate of the 5th lumbar vertebra [32–34]. This arrangement, which represents loading in vivo, allowed free rotation in all planes, thereby not restricting motion or causing displacement of the hemipelvis [33].

**Fig. 5:**
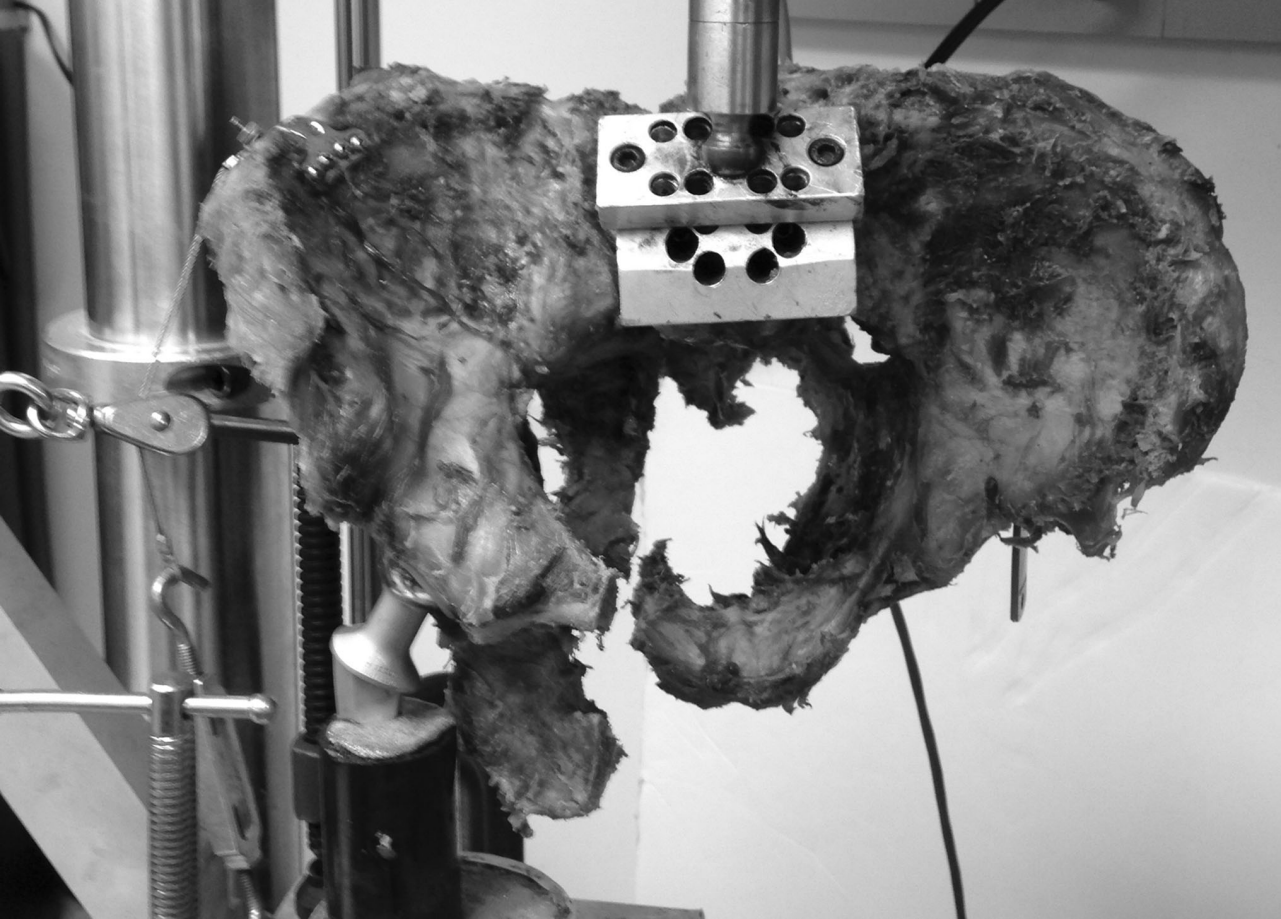
Pelvis following fixation failure

Subsequently, each pelvis was loaded at 250 N and cycled 100,000 times (equivalent to approximately 3 weeks of walking [35, 36]) at two cycles per second (2 Hz) and then loaded to failure (Fig. 5). The value of 250 N was selected, as it approximates the in vivo force applied through the spine during static single-limb stance [37, 38]. In addition, 250 N approximates 20 % of the load to failure in similar studies [34]. Therefore, we felt this applied force was sufficient but would allow 100,000 cycles of loading without causing gross failure of the fixation constructs or disrupting the positioning of the pelvis in the single-leg-stance setup. Vertical displacements were measured sequentially from the actuator at 25,000, 50,000, 75,000, and 100,000 cycles, and load-to-failure was recorded for each pelvis using the MTS software. To attain load to failure, a protocol was designed on the MTS software to lower the actuator on the MTS machine at a rate of 1 mm/s. As the load increased, progressive displacement resulting in fixation failure was expected to occur at the sacral osteotomy site. Load and displacement were recorded using MTS software. Failure was defined as the point on the load–displacement curve when force measurement declined rapidly toward zero and there was no further change in displacement [34].

Statistics were calculated using SPSS software (SPSS version 19; SPSS Chicago, IL, USA). The Mann–Whitney *U* test was used to compare bone density (as noted above), displacement, and load to failure of the two fixation groups. Freidman test was used for displacement comparisons within each group. The level of statistical significance was defined as *p* < 0.05.

## Results

All specimens completed 100,000 cycles with no gross evidence of construct failure. The progressive increase in displacement between each of these measured intervals was significantly different (*p* < 0.05) within both groups (Tables 1 and 2). In the group with supplemental long S1 iliosacral screw, mean displacements at the sacral osteotomy site at 25,000, 50,000, 75,000, and 100,000 were 14 ± 14.1, 18.5 ± 13.4, 20.7 ± 15.1, and 22.8 ± 15.7 mm, respectively (Table 1). In the group with a supplemental transsacral screw, mean displacements at the sacral osteotomy site at 25,000, 50,000, 75,000, and 100,000 cycles were 10.6 ± 3.9, 11.3 ± 4.3, 11.5 ± 4.4, and 12.3 ± 4.5 mm, respectively (Table 2). After undergoing 100,000 cycles at 250 N and each pelvis was loaded to failure, mean load to failure was 546 ± 174 N for the long iliosacral screw group and 635 ± 196 N for the transsacral screw group (Table 3).

**Table 1:** Posterior displacement for the long iliosacral group

Number of cycles	Displacement (in mm)				
	Minimum	Maximum	Mean	Standard deviation	*P* value*
25,000	4.8	38.8	14.0	14.1	–
50,000	9.2	42.0	18.5	13.4	0.025
75,000	9.4	47.3	20.7	15.1	0.025
100,000	9.5	49.3	22.8	15.7	0.025

* Freidman test

**Table 2:** Posterior displacement for the transsacral group

Number of cycles	Displacement (in mm)				
	Minimum	Maximum	Mean	Standard deviation	*P* value*
25,000	6.2	16.4	10.6	3.9	–
50,000	6.6	17.7	11.3	4.3	0.025
75,000	6.9	18.3	11.5	4.4	0.040
100,000	7.1	18.7	12.3	4.5	0.025

* Freidman test

**Table 3:** Load to failure for both groups

Group	Load to failure (in N)*			
	Minimum	Maximum	Mean	Standard deviation
Long iliosacral screws	398	849	546	174
Transsacral screws	414	931	635	196

* *P* value = 0.42, Mann–Whitney *U* test

Despite the fact that mean displacement values for the long iliosacral group were almost twice that of the transsacral group, analysis of displacements at 25,000, 50,000, 75,000, and 100,000 cycles showed no significant difference between groups (Table 4). In addition, there was no significant difference between groups in load to failure (Tables 3, 4). At the end of the study, gross inspection of each specimen revealed that all screws were intact without any obvious damage or deformity, and loss of fixation appeared to be caused by loss of surrounding S1 bone stock. A post hoc power analysis showed that with our sample size of 5 pelves in each group, our data had 24 % power for displacement and 10 % power for load to failure to detect a difference at *p* < 0.05.

**Table 4:** Comparison between groups

	Long iliosacral group	Transsacral group	*P* value*
Mean displacement at 25,000 cycles (in mm)	14.0	10.6	0.54
Mean displacement at 50,000 cycles (in mm)	18.5	11.3	0.42
Mean displacement at 75,000 cycles (in mm)	20.7	11.5	0.22
Mean displacement at 100,000 cycles (in mm)	22.8	12.3	0.15
Mean load to failure (in N)	546	635	0.42

* Mann–Whitney *U* test

## Discussion

Stabilization of posterior pelvic ring injuries with iliosacral screws inserted into the first sacral body is a commonly used technique [19, 20, 32, 39–41]. Yinger et al. and van Zwienen et al., in their biomechanical studies, showed that for a completely unstable pelvic ring injury, using two iliosacral screws increases rotational stiffness and load to failure [26, 28]. Consistent with these findings, two iliosacral screws inserted into S1, or one each into the S1 and S2 bodies, are used as a preferred method for fixation for these injuries [39]. However, this two-screw construct is clinically unreliable in some situations, especially with percutaneous fixation of unstable type C, zone 2, vertically oriented sacral fractures in which a residual gap exists at the fracture site [8].

Matta and Tornetta suggested that longer iliosacral screws might provide better fixation because they have greater resistance to toggle and are more resistant to vertical shear stress [39]. However, data to support this contention are wanting. A number of studies were unable to show any significant differences in fracture stability using different iliosacral screw lengths [8, 30]. Griffin et al., in a study evaluating percutaneous iliosacral screw fixation of 62 unstable type C injuries, used four different screw lengths: into the sacral body, to the level of the contralateral sacral foramen, to the contralateral sacral ala, and across the sacroiliac joint [8]. They reported that a vertical sacral fracture was the only statistically significant risk factor for fixation failure [8]. Tornetta et al. found that a construct using a standard iliosacral screw in combination with a transsacral screw performed no better than a standard two-screw construct [30]. However, Tabaie et al., in a biomechanical study with a design similar to ours, compared standard iliosacral screws to a novel locked transsacral screw construct and reported significantly improved fixation using the transsacral locked method [34].

 The purpose of our study was to assess the potential improvement of fixation using one of two alternative long-screw fixation options: a transsacral or a long iliosacral implant. In order to create an “extreme” condition, osteopenic/osteoporotic pelvic specimens were used, and the anterior fractures were not fixed. This allowed us to focus directly on the posterior fixation in a model at the greatest risk for fixation failure. In addition, to maximize vertical shear and minimize compression across the posterior pelvic arch, a single-limb-stance model was used [32].

Our study has a number of limitations. First, despite the use of nonparametric statistics, our failure to show a difference between the two groups may be type 2 error due to the relatively small sample size and lack of sufficient statistical power. Our selection of five specimens in each group was based on the findings of Tabaie et al. [34]. However, post hoc power analysis indicated low statistical power, which was due to the relatively large standard deviations in our results (Tables 1–3). Therefore, despite the fact that the mean displacement values for the long iliosacral group were almost twice that of the transsacral group, there were no significant differences between groups. These large variations from specimen to specimen were not found by Tabaie et al. and might represent greater variability in our specimens or testing apparatus. However, it is interesting to note that comparison of our raw data to those of Tabaie et al. revealed that the locked transsacral screw construct reported by Tabaie et al. has a significantly greater load to failure than our two fixation constructs or the short iliosacral construct tested by Tabaie et al. (Table 5). In any case, the issue of specimen-to-specimen variability, compounded by a relatively small sample size, is a common problem in biomechanical studies [26, 30, 34, 37, 38, 42]. Perhaps the differences between our study groups would have reached statistical significance with a much larger number of specimens. Second, using embalmed rather than fresh-frozen cadaver specimens is another potential limitation. However, Comstock et al. used embalmed cadaver specimens in a biomechanical evaluation of fixation of the posterior pelvic ring and found results comparable with studies performed with fresh-frozen specimens [42]. More recently, van Zwienen et al. found embalmed pelvic specimens to be satisfactory for biomechanical evaluation of unstable pelvic ring injuries [38].

**Table 5:** Comparison of load to failure for long iliosacral, transsacral, short iliosacral, and locked transsacral screw constructs

Group	Load to failure (in N)				*P* value in comparison with the other screw constructs
	Minimum	Maximum	Mean	Standard deviation	
Locked transsacral screws (from [34])*	929	1201	1056	118	
Long iliosacral screws	398	849	546	174	0.008
Transsacral screws	414	931	635	196	0.016
Short iliosacral screws (from [34])	798	874	825	31	0.008

* Significantly different from the other three groups (multiple comparisons using Mann–Whitney *U* test with Bonferroni correction)

Although there have been a number of studies comparing standard iliosacral screws with longer screw constructs, we know of no study directly comparing these longer screw methods. Tornetta et al. described the concept of different modes of failure [30], reporting that standard screws cut through the sacrum while long screws bent, indicating that the long screw was better anchored at its distal end [30]. However, in our study, after applying load to failure, none of the screws in either group were bent or broken. Our mode of failure was at the S1 body and alar bone stock. This difference may be related to dissimilarities in design between the two studies: ours using a fracture-gap single-stance model; theirs using anatomic reduction in a bilateral-stance model.

Although intuitively a transsacral screw may seem to be more advantageous than a long iliosacral screw in conveying additional stability to a type C, zone 2, vertically unstable sacral fracture fixation construct, we were not able to identify any biomechanical advantage of one fixation method over the other. Further study with a larger number of samples may be required to more accurately compare screw configuration in these injuries.
